# Habitat fragmentation is linked to cascading effects on soil functioning and CO_2_ emissions in Mediterranean holm-oak-forests

**DOI:** 10.7717/peerj.5857

**Published:** 2018-10-30

**Authors:** Dulce Flores-Rentería, Ana Rincón, Teresa Morán-López, Ana-Maria Hereş, Leticia Pérez-Izquierdo, Fernando Valladares, Jorge Curiel Yuste

**Affiliations:** 1 Group of Sustainability of Natural Resources and Energy, CONACYT—CINVESTAV Unidad Saltillo, Ramos Arizpe, Coahuila, Mexico; 2 LINCGlobal, Department of Biogeography and Global Change, Museo Nacional de Ciencias Naturales (MNCN), Spanish Scientific Council (CSIC), Madrid, Spain; 3 Department of Soil, Plant and Environmental Quality, Instituto de Ciencias Agrarias (ICA), Spanish Scientific Council (CSIC), Madrid, Spain; 4 Ecotono Lab, INIBIOMA, CRUB, Universidad Nacional del Comahue, Bariloche, Río Negro, Argentina; 5 Department of Forest Sciences, Transilvania University of Brasov, Brasov, Romania; 6 BC3-Basque Centre for Climate Change, Scientific Campus of the University of the Basque Country, Leioa, Spain; 7 Department of Biology and Geology, Universidad Rey Juan Carlos, Móstoles, Madrid, Spain; 8 IKERBASQUE, Basque Foundation for Science, Bilbao, Bizkaia, Spain

**Keywords:** Structural equation models, *Quercus ilex*, Soil functioning, Forest fragmentation

## Abstract

We studied key mechanisms and drivers of soil functioning by analyzing soil respiration and enzymatic activity in Mediterranean holm oak forest fragments with different influence of the agricultural matrix. For this, structural equation models (SEM) were built including data on soil abiotic (moisture, temperature, organic matter, pH, nutrients), biotic (microbial biomass, bacterial and fungal richness), and tree-structure-related (basal area) as explanatory variables of soil enzymatic activity and respiration. Our results show that increased tree growth induced by forest fragmentation in scenarios of high agricultural matrix influence triggered a cascade of causal-effect relations, affecting soil functioning. On the one hand, soil enzymatic activity was strongly stimulated by the abiotic (changes in pH and microclimate) and biotic (microbial biomass) modifications of the soil environment arising from the increased tree size and subsequent soil organic matter accumulation. Soil CO_2_ emissions (soil respiration), which integrate releases from all the biological activity occurring in soils (autotrophic and heterotrophic components), were mainly affected by the abiotic (moisture, temperature) modifications of the soil environment caused by trees. These results, therefore, suggest that the increasing fragmentation of forests may profoundly impact the functioning of the plant-soil-microbial system, with important effects over soil CO_2_ emissions and nutrient cycling at the ecosystem level. Forest fragmentation is thus revealed as a key albeit neglected factor for accurate estimations of soil carbon dynamics under global change scenarios.

## Introduction

Mediterranean forests have been intensively transformed by humans during centuries to obtain resources (e.g., water and nutrients). Regarding soils, several studies in fragmented Mediterranean forests have shown that habitat fragmentation induces changes in soil microclimatic environmental conditions (e.g., soil water, organic matter and nutrient contents), which causes modifications in the structure and metabolic performance of the microbial communities (Flores-Rentería et al., 2015; Flores-Rentería et al., 2016; Lázaro-Nogal et al., 2012) with consequences for soil nutrient cycling. In the long term, soil legacies from fragmented forest may even compromise the capacity of drought-tolerant plant provenances to survive environmental drought stress (Flores-Rentería et al., 2018). Nevertheless, much remains to be known about the ecological mechanisms affecting soil functioning (e.g., enzymatic activities) and how this may impact total soil respiration (*R*_*s*_). The potential effects of forest fragmentation on soil functioning are of special interest, given that soils are the most important long-term carbon reservoir in terrestrial ecosystems, representing the main outgoing flux of CO_2_ to the atmosphere (Schlesinger & Andrews, 2000), and that land use changes are considered a major global change driver (Sala et al., 2000). Hence, quantifying and unraveling forest fragmentation effects on soil organic matter decomposition and subsequent soil CO_2_ emissions is crucial to understand how anthropogenic activities are modifying the potential capacity of terrestrial systems to sequester C and mitigate global warming.

Forest fragmentation may alter ecosystem functioning at many levels since solar radiation, wind, water, and temperature are significantly modified in the remaining forest fragments (Saunders, Hobbs & Margules, 1991). In general, forest edges are characterized by higher radiation and wind exposure, resulting in higher evapotranspiration rates (Murcia, 1995). Beyond changes in micro-climatic conditions, forest fragmentation can be followed by changes in vegetation structure. In particular, in Mediterranean forests, reduction of competition for water resources at forest edges has been linked to bigger trees with increased tree acorn production and water potential (Morán-López et al., 2016). Increased tree size is expected to result in higher organic matter availability (Christensen, 2001). Additionally, since soil enzymatic activity can be considerably affected by net primary productivity (Pérez-Izquierdo et al., 2017), it is predictable that potential effects of habitat fragmentation over tree growth may also impact functions of particular enzymes involved in key soil metabolic pathways (e.g., C, N and P cycling). Concomitantly to changes related to habitat loss, the influence of the surrounding agricultural matrix can be non-negligible. For instance, agricultural matrix has a positive impact in the amount of soil nutrients, which in turn modifies soil microbial taxonomic and functional diversity in a landscape scale (Flores-Rentería et al., 2015; Flores-Rentería et al., 2016). If key environmental factors for soil respiration and enzymatic activities are modified in fragmented areas, we would expect that fragmentation effects can be explained in the light of the ecological mechanisms involved in soil functioning.

However, how all these factors affect the soil system functioning (as nutrient cycling and CO_2_ emissions) remains uncovered. Despite the well-known fact that changes in *R*_*S*_, particularly in semi-arid systems, can result in strong modifications of the ecosystem source–sink capacity (Baldocchi, Tang & Xu, 2006; Wang & Epstein, 2013) and that most Mediterranean ecosystems have historically suffered strong fragmentation due to extensive agricultural practices (Matesanz, Escudero & Valladares, 2009), there are very few studies that have evaluated how forest fragmentation affects this important terrestrial outgoing CO_2_ flux and its different components.

Our objective here was to investigate in depth the ecological mechanisms controlling fragmentation effects on soil enzymatic activity and CO_2_ emissions. For this purpose we integrated all the information previously generated by our research group, taking into account how forest fragmentation affected forest structural (tree size and proximity), microbial (microbial biomass and richness), and environmental (microclimate, soil chemistry) factors and how these changes could trigger direct and indirect effects on soil functioning.

We hypothesized that increased influence of the agricultural matrix in forest fragments would promote tree growth. This, would result in higher organic matter input to the soil with impacts on chemistry and microclimatic conditions, which would promote microbial respiration and enzymatic activity in fragmented areas. Therefore, we expected that forest fragmentation effects on soil functioning (*R*_*s*_ and enzymatic activity) would be mediated by matrix influence effects on local environmental conditions modulated by tree size.

## Material and Methods

### Study area

The study area was located near Quintanar de la Orden (39°30′–39°35′N, 02°47′–02°59′W; 870 a.s.l.), in Toledo, southeastern Spain. This area has a Mesomediterranean climate characterized by 434 mm of mean annual precipitation and 14 °C of mean annual temperature (Ninyerola, Pons & Roure, 2005), with a pronounced summer drought, usually lasting from July to September. The landscape, a former predominant holm oak Mediterranean forest, is currently dominated by cereal and legume croplands, with scattered grape crops that complete the mosaic. The original forests are now highly fragmented in a variety of patch sizes, covering only the 28% of their original extent (Díaz & Alonso, 2003). The dominant tree is the holm oak (*Quercus ilex* L. ssp. ballota (Desf.) Samp), while the understory is mainly composed by shrubs of Kermes oak (*Quercus coccifera* L.) and, to a lesser extent, by species of *Genista*, *Asparagus*, and *Rhamnus* (for a full description of the study area see: Díaz & Alonso, 2003; Flores-Rentería et al., 2015; Flores-Rentería et al., 2016).

### Experimental design and sampling

A total of three large (>10 ha; with 121 stems per ha on average) and five small (<0.5 ha; with at least three trees) forest fragments within an area of 1,000 ha, separated by a minimum of 50 m (to avoid spatial dependence) to a maximum of 8 km, were studied. Prevalent soils were Cambisols (calcics) (WRB, 2007), with sandy loam texture (17–39–44%, sand-silt-clay, respectively).

There is strong evidence that the exposure of the edges of fragmented forest causes changes in the abiotic and biotic conditions in comparison with forest interiors (Fischer & Lindenmayer, 2007; Flores-Rentería et al., 2015; Flores-Rentería et al., 2016; Murcia, 1995), while small fragments consist effectively only of edge habitat (Young & Mitchell, 1994). For instance, in the study area, forest interiors show higher intraspecific competition (measured as the proportion of area within a radius of 20 m from focal trees covered by other canopies), in comparison with edges and small fragments (0.46  ± 0.04, 0.36  ± 0.03, and 0.27  ± 0.14, interior, edges and small fragments, respectively) (Morán-López et al., 2016). Thus, we defined the influence of the agricultural matrix on forest fragments by the factor “matrix influence” with three levels: (1) low influence, at the interior of large fragments (at least 30 m from the forest edge; coded as “forest interior”); (2) mid influence, at the edges of large fragments (coded as “forest edge”); and (3) high influence, in small fragments (coded as “small fragments”). For each of the three large fragments, we selected five holm oak trees in the forest interior and five trees at the forest edge, while for each of the five small fragments we selected three holm oak trees (15 trees per fragmentation level), resulting in a total of 45 selected trees. For each of the focal trees, two coverage-sampling points were established: one under canopy (half way to the stem) and the other in open areas (with visible clear sky above, at least 1.5 m to another canopy), resulting in a total of 90 soil samples (see Flores-Rentería et al., 2016).

### Field measurements

The field campaign was conducted in spring 2013, during the rainy growing season in Mediterranean ecosystems, when temperature and moisture were not limiting factors neither for plant growth nor for soil microbial functioning. Daily maximum temperature during the sampling days was of 24  ± 1.5  °C, with 15  ± 1.5 °C in average, and no precipitation was recorded.

Soil respiration (*R*_*s*_) was measured at each sampling point with a portable dynamic closed chamber (SRC-1; P Systems, Amesbury, MA, USA) connected to an infrared gas analyzer (EGM-4; P Systems, Amesbury, MA). The soil chamber was placed on 90 external PVC collars (5 cm depth × 10 cm diameter) temporarily inserted to a depth of 3 cm into the soil 24 h before *R*_*s*_ measurements to minimize the impact of its insertion (e.g., underestimate *R*_*s*_ from roots) (Heinemeyer et al., 2011). *R*_*s*_ measurements were carried out at maximum daily soil activity (13:30–16:30 h) (Matías, Castro & Zamora, 2011), during three consecutive days (each sample point was measured one time). Immediately after *R*_*s*_ was measured, soil temperature and soil moisture were recorded at 10 cm depth by using a wireless multilogger thermometer (OMEGA, Norwalk, CT, USA), and a time domain reflectometer (TDR 300, Spectrum technologies, Illinois, USA), respectively. Then, soil cores (2 cm in diameter) were taken from a depth of 0–15 cm. Soils were sieved (<2 mm) and stored at 4 °C for later analyses.

A tree influence index was calculated for each sampling point, to take into account the influence of tree size and proximity according to the following formula:

\begin{eqnarray*}\text{Tree influence index}  \left( \mathrm{Tii} \right) = \frac{\mathrm{BS}}{\mathrm{DT}} . \end{eqnarray*}

Where BS stands for the basal area measured at 25 cm from the ground (D_25_), and based on stems ≥3 cm of diameter; and DT, the distance from the trunk, which was established as half the radio of the canopy cover in the case of the under-canopy points, and at 1.5 m from the edge of the canopy in the case of the open-areas. Additionally, height and canopy projection were measured for each holm oak tree. Tree basal area was selected to calculate the index, given its recognized direct relationship with soil functioning (Barba et al., 2013; Søe & Buchmann, 2005).

### Soil properties

Soil moisture was determined by weight losses of 20 g samples oven-dried at 105 °C for 48 h. Total carbon (C) and nitrogen (N) contents were measured on air-dried soil samples, using a C:N elemental analyzer (Flash EA 1112 Series; Thermo Fisher Scientific, Waltham, MA, USA). Soil organic matter (SOM) was assessed by loss on ignition at 400 °C for 4 h (Ball, 1964). Microbial biomass C content was determined by the chloroform fumigation - extraction method modified by Gregorich et al. (1990).

### Soil microbial community richness

The structure of soil bacterial and fungal communities was assessed by the DNA community fingerprinting technique of denaturing gradient gel electrophoresis (DGGE). The universal primers 338F/518R were used for amplification of the bacterial 16S rRNA gene (Muyzer, De Waal & Uitterlinden, 1993). In the case of fungi, the internal transcribed spacer nrDNA region ITS-1 was PCR-amplified using the primer pair ITS1-F/ITS2 (Gardes & Bruns, 1993). A GC clamp was respectively added to the 5′ end of forward bacterial (338F) and fungal (ITS1-F) primers to stabilize the melting behavior of the DNA fragments (Muyzer, De Waal & Uitterlinden, 1993). DGGE was carried out in gradients of 10–50% for fungi (Anderson, Campbell & Prosser, 2003) and 30–60% for bacteria (Grossman et al., 2010), with the concentrations of 7 M urea and 40% formamide (v/v) for the 100% denaturant. Electrophoreses were run at 60 °C 75 V for 16 h. Each band of the DGGE profile was hereafter referred to as an operational taxonomic unit (OTU). Gel bands were analyzed by using internal reference bands and known reference markers loaded in lanes at either side of the gel. The number of bands in a particular sample was considered comparative proxies of richness (S) of fungal or bacterial OTUs, respectively (Cleary et al., 2012). Similar analysis of DGGE banding patterns have been previously used in other studies (Anderson, Ellingsen & McArdle, 2006; Cleary et al., 2012; Flores-Rentería et al., 2015; Flores-Rentería et al., 2016; Suzuki et al., 2012; Vaz-Moreira et al., 2013).

### Soil enzymatic activity

To characterize soil heterotrophic metabolism, we determined the polysaccharide-specific hydrolytic enzymes *β*-glucosidase and chitinase, and the phosphorus-mineralizing acid phosphatase in fresh soil samples. Enzymatic assays were based on methylumbelliferone (MU) (fluorogenic substrate) release upon cleavage by enzymes (Mathieu et al., 2013): MU-*β*-d-glucopyranoside (MU-G) for *β*-glucosidase (EC 3.2.1.3), MU-N-acetyl- *β*-glucosaminide (MU-Q) for chitinase (EC 3.2.1.14), and MU-phosphate free acid (MU-P) for acid phosphatase (EC 3.1.3.2). Stocks of 5 mM enzyme solutions were prepared in methoxy-ethanol, and enzyme substrates were diluted in sterile ultra-pure water to a final concentration of 800 µM for MU-P and 500 µM for MU-G and MU-Q assays. Stocks and calibration solutions, as well as diluted substrates were kept at −20 °C in the dark. Fluorogenic assays were performed by mixing 200 µl of soil supernatant (soil incubated over night with Tris-acetate buffer 10 mM, pH 4.5 in a horizontal shaker at 25 °C and 100 rpm), and 50 µl of the corresponding substrate with a final volume of 250 µl per well, using black 96-well microplates. Controls with soil supernatant heated at 100 °C for 10 min were also conducted separately for each sample. All reactions were performed at room temperature, applying a stirring of 500 rpm, in the dark and at different incubation times depending on the enzymatic test: 15 min (MU-P), 40 min (MU-G), and 60 min (MU-Q). After incubation, microplates were spin (3,000 rpm for 3 min), and 100 µl of the reaction mix was added to 100 µl of stopping buffer (Tris 1 M, pH 10–11). Measurements were carried out with a Victor3 microplate reader (Perkin-Elmer Life Sciences, Waltham, MA, USA), at 355/460 nm excitation-emission wavelengths. Experimental calibrations of known MU concentrations were performed, allowing estimating each enzymatic activity by extrapolating well fluorescence signals on the respective calibration regression lines. Blanks with buffer and fluorogenic substrates related to auto-fluorescence, and controls were subtracted from all measures. All enzymatic activities are expressed in pmol min^−1^ mg^−1^ of dry soil.

### Data analysis

Prior to statistical analyses, all variables were tested for normality, and log transformations were applied to meet variance homoscedasticity when required. Besides, a principal component analysis (PCA) was conducted to reduce the dimensionality of soil nutrient data into two linear axes explaining the maximum amount of variance. Because our focal trees were spatially arranged within fragments, we evaluated if they could be considered as independent replicates since our dataset could have a spatial autocorrelation structure. Before modeling, we checked for spatial autocorrelation in soil respiration and enzymatic activity (glucosidase, phosphatase and chitinase). To do so, we used Moran I function (ape library, Gittleman & Kot, 1990). We found no spatial autocorrelations in our response variables, and hence, we considered our sampling points to be independent replicates. After our analysis, we performed a Moran test on the ANOVA model residuals and found no significant effects. In the case of SEM models, the standardized residuals were always <2.58, indicating non-significant discrepancy among variables (Grace, 2006). Thus, model residuals supported our initial assumption of sample independence. Soil functional, biotic and abiotic environmental variables were analyzed by two-way Analysis of Variance (ANOVA) considering the factors matrix influence (low, medium, high) and coverage (under canopy, open areas). Since coverage had a predominant effect on soil functioning, we assessed coverage and matrix effects separately. To do so, we firstly quantified the effects of tree cover for a given matrix level, and, secondly, the effects of matrix influence for a given coverage. For this purpose, we used one-way ANOVA. Tukey’s HSD were used as post hoc test (*p* < 0.05).

Structural equation models (SEMs) were used to test the direct and indirect influence of both biotic and abiotic factors on measured soil microbial functioning variables *R*_*s*_ and enzymatic activity (as latent variable). The latent variables are those not directly observed (i.e., enzymatic activity) but rather inferred from other observed variables, directly measured, denominated indicators (i.e.,  *β*-glucosidase, chitinase, and phosphatase). Our models considered a complete set of hypotheses based on literature, previous exploratory analyses and our own experience (Flores-Rentería et al., 2015; Flores-Rentería et al., 2016). In short, we hypothesized that matrix influence would promote nutrient input and tree growth (Flores-Rentería et al., 2015; Morán-López et al., 2016). Increased tree size would result in higher organic matter available in the soil (SOM), which in turn would modify abiotic conditions (i.e., increased moisture) (Abu-Hamdeh, 2001). These new abiotic conditions combined with increased SOM would result in higher respiration rates and overall enzymatic activity (Pérez-Izquierdo et al., 2017; Schlesinger & Andrews, 2000). Several models were run, and the best-fitted ones were finally selected according to the covariance proximity between observed and expected data (goodness-of-fit *χ*^2^). Standardized path coefficients were estimated by using the maximum likelihood algorithm (Shipley, 2002). The degree of fit between observed and expected covariance structures was assessed by root mean square error of approximation statistic (RMSEA) (Steiger, 1990). RMSEA values <0.08 indicate a good fit, between 0.08 and 0.10 provide a moderate fit, and >0.10 suggest a poor fit (Maccallum, Browne & Sugawara, 1996). Model fit to data was additionally evaluated by the goodness-of-fit index (GFI) and the Bentler and Bonett’s normed-fit index (NFI), both with values ranging between 0 and 1, and those >0.9 indicating an acceptable fit (Iriondo, Albert & Escudero, 2003). All statistical analyses were performed by using SPSS® and SPSS® AMOS 20.0 software’s (IBM Corporation Software Group, Somers, NY, USA).

## Results

### Soil characteristics

Soil biotic and abiotic variables were strongly influenced by the canopy cover (Table 1). Under canopy, significantly higher values of soil moisture, SOM, and lower values of soil temperature and pH, were found compared to open areas (Table 1; Table S1). Matrix effects were related to soil temperature and moisture. Soil temperature tended to be lower in forest edges (intermediate influence), both under canopy and in open areas. In the case of moisture, matrix effects were mediated by coverage. Only in open areas it was significantly affected by the agricultural matrix, with the highest values recorded at forest edges (Table 1; Table S1). Organic matter and pH were not significantly affected by matrix influence. In areas with higher matrix influence, trees were higher (basal area, height and canopy projection), which resulted in an overall higher tree influence (Fig. S1). According to the PCA of soil variables, the PC1 axis reflected a gradient of nutrient availability related with the degree of forest fragmentation, with higher amounts of C, N, P, Ca^2+^, S, and Mg^2+^ in small fragments and at forest edges than in forest interiors (Fig. S2). Regarding biotic variables, tree cover affected microbial biomass but did not affect community composition (bacterial and fungal richness). In contrast, matrix influence did not modify microbial biomass but affected bacterial richness, with more OTUs at small fragments and forest edges in comparison with the forest interiors (Table 1, Table S1).

**Table 1:** Characteristics of soil and trees in fragments with three matrix influence levels (low, forest interior; mid, forest edge; and high, small fragment) of holm oak forests in Spain.

**Cover**	**Under canopy**			**Open areas**		
**Matrix influence**	**Forest interior**	**Forest edge**	**Small fragments**	**Forest interior**	**Forest edge**	**Small fragments**
**ABIOTIC VARIABLES**						
**Soil organic matter (%)**	7.7 ± 0.5^a,A^	9.9 ± 0.5^a,A^	16.1 ± 1.0^a,A^	3.5 ± 0.3^a,B^	4.4 ± 0.4^a,B^	4.7 ± 0.5^a,B^
**pH**	8.0 ± 0.1^a,B^	8.0 ± 0.1^a,B^	7.9 ± 0.1^a,B^	8.2 ± 0.1^a,A^	8.2 ± 0.1^a,A^	8.2 ± 0.1^a,A^
**Soil moisture (%)**	13.4 ± 0.5^a,A^	15.5 ± 0.7^a,A^	19.0 ± 0.9^a,A^	7.2 ± 0.3^b,B^	10.3 ± 0.5^a,B^	8.4 ± 0.4^ab,B^
**Soil temperature (°C)**	18.8 ± 0.3^a,B^	17.6 ± 0.3^b,B^	18.1 ± 0.3^ab,B^	26.1 ± 0.4^a,A^	22.7 ± 0.5^b,A^	26.3 ± 0.4^a,A^
**BIOTIC VARIABLES**						
**Tree influence index (Tii)**	318.1 ± 3.1^b,A^	603.3 ± 4.2^a,A^	572.6 ± 4.9^a,A^	109.1 ± 1.8^b,B^	239.2 ± 2.8^a,B^	219.0 ± 3.1^a,B^
**Bacterial richness (S)**	34 ± 0.52^b,A^	36.93 ± 0.38^a,A^	37.53 ± 0.52^a,A^	32.6 ± 0.6^b,A^	35.27 ± 0.5^a,A^	36.8 ± 0.47^a,A^
**Fungal richness (S)**	29.4 ± 0.47^n.s.^	28.8 ± 0.32^n.s.^	27.93 ± 0.37^n.s.^	29.73 ± 0.45^n.s.^	29.27 ± 0.37^n.s.^	28.73 ± 0.31^n.s.^
**Microbial biomass (mg C kg^−1^)**	1170.7 ± 4.9^a,A^	1576.3 ± 5.9^a,A^	2438.0 ± 12.7^a,A^	635.4 ± 3.6^a,B^	810.9 ± 4.3^a,B^	769.0 ± 4.8^a,B^

**Notes.**

Capital letters represent differences among tree cover for a given matrix influence level, one way-ANOVA (*p* < 0.05, *n* = 30), while lowercase letters represent differences among matrix influence for a given tree cover (under canopy or open areas), one way-ANOVA (*p* < 0.05, *n* = 45). Data are means ± standard error.

### Matrix and tree cover influence in soil functioning

**Figure 1:**
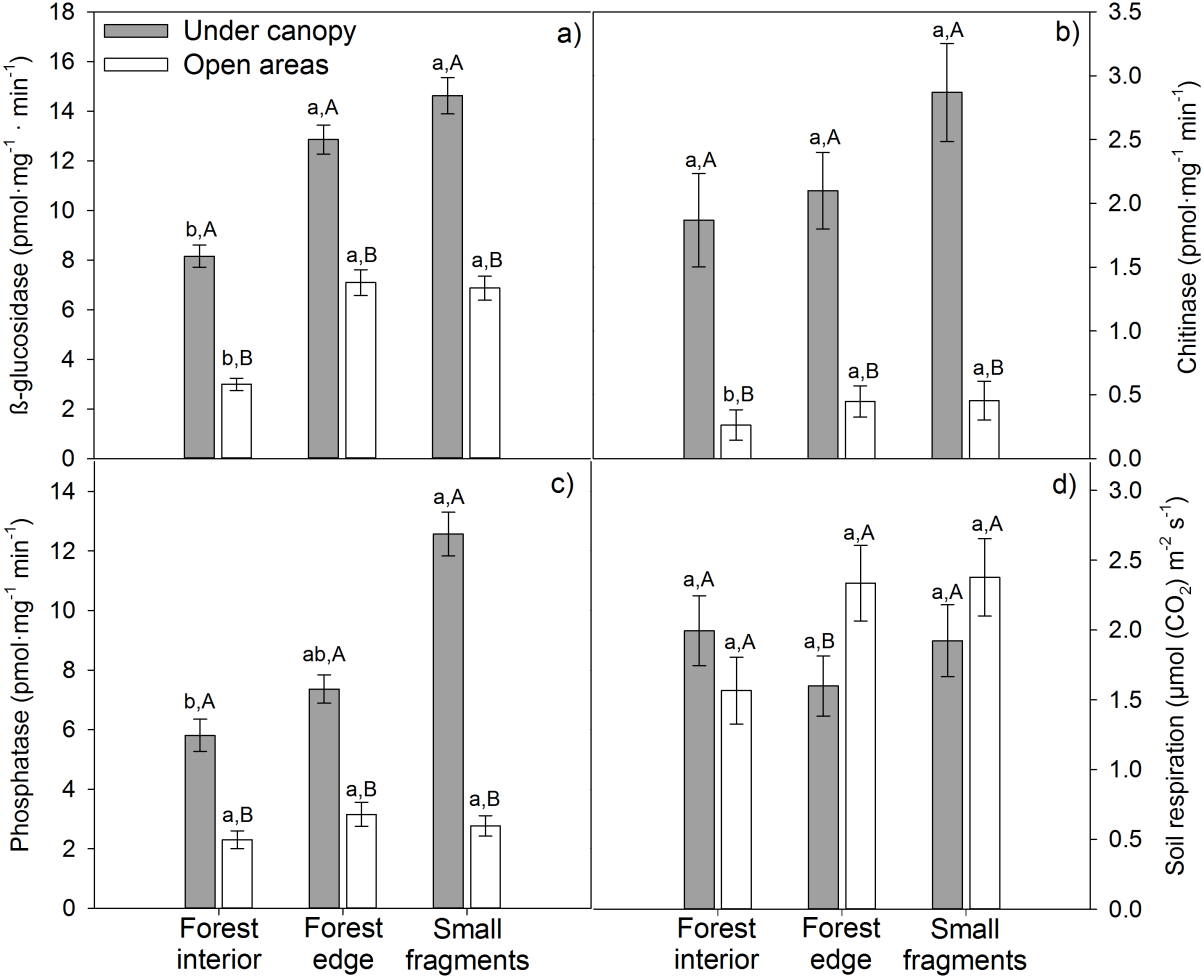
Enzymatic activities (A) glucosidase, (B) chitinase, (C) phosphatase and (D) respiration (*R*_*S*_) of soils from three agricultural matrix influence levels of holm oak forests in Spain. Coverage is represented by different colors: gray = under canopy (UC); white = open areas (OA). Matrix influence is presented at three levels (low, forest interior; mid, forest edge; and high, small fragment). Capital letters depict differences among tree cover for a given matrix influence level, one way-ANOVA (*p* < 0.05, *n* = 30), while lowercase letters represent differences among matrix influence for a given tree cover (under canopy or open areas), one way-ANOVA (*p* < 0.05, *n* = 45). Data are means ± standard error.

Tree cover significantly and positively affected all enzymatic activities, independently of the level of influence of the matrix (Table S1; Figs. 1A–1C). Maximum *β*-glucosidase (14.62 pmol mg^−1^ min^−1^), chitinase (2.87 pmol mg^−1^ min^−1^) and phosphatase (12.57 pmol mg^−1^ min^−1^) activities were obtained under canopy in small fragments (Figs. 1A–1C). Regarding matrix influence, its effects depended on the particular enzymatic activity. *β*-glucosidase activity was significantly lower at forest interiors, both under canopy and in open areas (Fig. 1A). Nonetheless, matrix effects on the rest of enzymatic activities were modulated by tree cover. While in open areas chitinase and phosphatase activities were unaffected by matrix influence, under canopy, these enzymatic activities were significantly higher in small fragments (Figs. 1B–1C). Fragmentation effects on soil respiration patterns were weaker. The agricultural matrix did not have a significant effect on *R*_*s*_ in any case, while tree cover did at forest edges, where *R*_*s*_ was significantly higher in open areas than under canopy (Fig. 1D).

**Figure 2:**
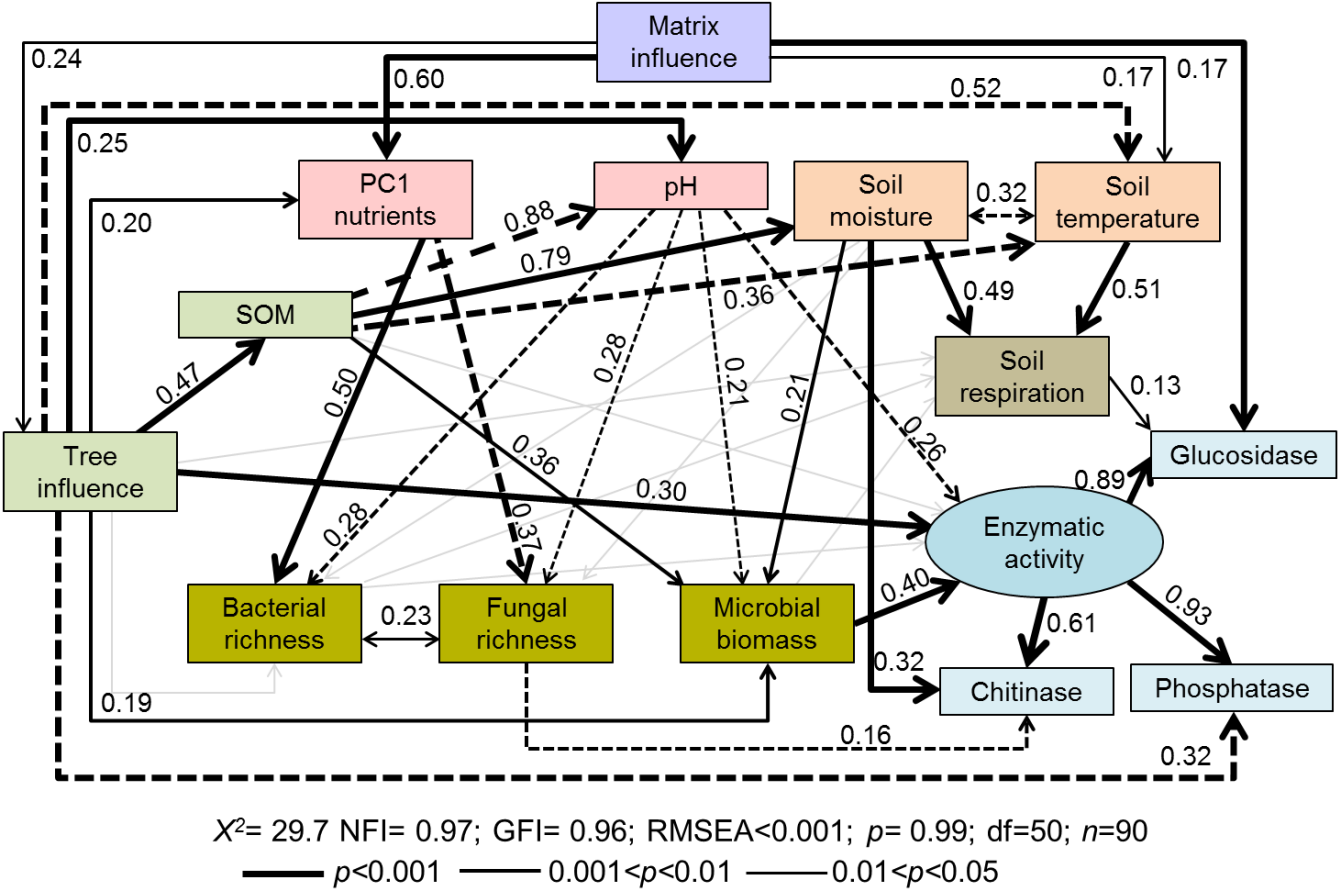
Path diagrams representing hypothesized causal relationships among the tree influence (proxy by tree size), biotic and abiotic variables, soil respiration and soil enzymatic activity (indicated by *β*-glucosidase, chitinase, and phosphatase). Arrows depict causal relationships: positive and negative effects are indicated by solid and dashed lines respectively, with numbers indicating standardized estimated regression weights (SRW). Arrow widths are proportional to significance values according to the legend. Paths with coefficients non-significant are in gray.

The structural-equation model proposed for soil functioning (Fig. 2) provided a good fit as indicated by the non-significant *f* value (*χ*^2^ = 29.7; *p* = 0.99) and by the goodness-of-fit indices (RMSEA < 0.001; NFI and GFI > 0.96). The indicators of the proposed latent variable of enzymatic activity (*β*-glucosidase, chitinase, and phosphatase) showed a good fit. All tested soil enzymatic activities showed high maximum likelihood estimates: phosphatase 0.93, *β*-glucosidase 0.89, and chitinase 0.61. Squared multiple relations also exhibited high amounts of explained variance of the different variables included in the model, especially for the soil functioning indicators: *β*-glucosidase (89.8%), soil enzymatic activity (82%), chitinase (77.1%), phosphatase (60.1%) and *R*_*s*_ (17.7%) (Table 2).

The proposed general fitted model showed that the agricultural matrix influence had a positive impact on PC1 axis (i.e., soil nutrients), tree influence, and soil temperature (Fig. 2). Tree influence significantly and directly affected many different soil properties such as soil temperature (negatively), SOM amount, nutrients, pH and microbial biomass (positively) (Fig. 2), which modified soil respiration and enzymatic activities.

According to standardized regression weights, microbial biomass was directly affected by SOM (positive), soil moisture (positive) and pH (negative) (Fig. 2). Soil enzymatic activity was mainly driven by microbial biomass, followed by the tree influence (positive) and soil pH (negative) (Fig. 2; Table 3 direct effects). Specific enzymatic activities were regulated by different pathways. *β*-glucosidase activity was modulated by matrix influence and soil respiration. Chitinase activity was positively related to soil moisture but negatively related to fungal richness. In the case of phosphatase, it was negatively directly affected by tree influence though total effects of this variable turned positive (*T* = 0.24, Table 2). Contrary to our expectations, neither SOM amount or nutrient input directly affected overall enzymatic activity (Fig. 2). However, in the case of SOM some indirect paths were observed, i.e., through its effects on microbial biomass and soil pH.

**Table 2:** Standardized total (T), direct (D) and indirect (I) effects of biotic and abiotic variables descriptive of the plant-soil system on its functional response of the structural equation model (See Fig. 2). Functional response estimated as CO_2_ emissions (*R*_*s*_, soil respiration) and nutrient cycling (enzymes), based on standardized regression weights (SRW). Significant direct effects are noted in bold (*n* = 90).

	***R*_*s*_**			**Enzymatic activity**			***β*-glucosidase**			**Chitinase**			**Phosphatase**		
	**T**	**D**	**I**	**T**	**D**	**I**	**T**	**D**	**I**	**T**	**D**	**I**	**T**	**D**	**I**
**ABIOTIC VARIABLES**															
**SOM**	0.127	0	0.127	0.646	0.135	0.511	0.609	0	0.609	0.638	0	0.638	0.613	0	0.613
**PC1 nutrients**	0.054	0	0.054	0.036	0	0.036	0.04	0	0.04	0.075	0	0.075	0.035	0	0.035
**pH**	−0.004	0	−0.004	−0.355	**−0.253**	−0.102	−0.326	0	−0.326	−0.169	0	−0.169	−0.336	0	−0.336
**Soil moisture**	0.436	**0.484**	−0.048	0.072	0	0.072	0.122	0	0.122	0.41	**0.316**	0.074	0.068	0	0.068
**Soil temperature**	0.507	**0.507**	0	0	0	0	0.065	0	0.065	0	0	0	0	0	0
**BIOTIC VARIABLES**															
**Matrix influence**	0.127	0	0.127	0.157	0	0.157	0.327	**0.174**	0.153	0.163	0	0.163	0.068	0	0.068
**Tree influence**	0.063	0.243	−0.18	0.622	**0.306**	0.315	0.578	0	0.578	0.517	0	0.517	0.243	**−0.317**	0.59
**Bacterial richness**	0.108	0.108	0	0.072	0.072	0	0.08	0	0.08	0.043	0	0.043	0.069	0	0.069
**Fungal richness**	0	0	0	0	0	0	0	0	0	−0.148	**−0.158**	0	0	0	0
**Microbial biomass**	−0.13	−0.13	0	0.396	**0.396**	0	0.346	0	0.346	0.235	0	0.235	0.376	0	0.376

**Notes.**

Matrix influence agricultural matrix influence SOM soil organic matter

**Table 3:** Rates of explained variation of different components of the edaphic environment as influenced by their direct or indirect causal relationships of the structural equation model (See Fig. 2).

	**Estimate (%)**
*Microclimate*	
Soil moisture (%)	63.0
Soil temperature (°C)	54.4
*Abiotic properties*	
Soil organic matter (%)	21.9
PC1 nutrients of the PCA	45.2
pH	62.4
*Biotic properties*	
Microbial biomass (mg C kg^−1^)	65.7
Bacterial richness (S)	37.7
Fungal richness (S)	19.4
*Soil functioning*	
*Rs* (µmol (CO_2_) m^2^ s^−1^)	17.7
Enzyme activity (latent variable)	81.6
Chitinase (pmol min^−1^ mg^−1^)	77.1
Phosphatase (pmol min^−1^ mg^−1^)	60.1
*β*-glucosidase (pmol min^−1^ mg^−1^)	89.8

**Notes.**

*R*_*S*_ soil respiration

Soil respiration was positively and directly affected by the environmental factors, temperature and moisture, which were in turn indirectly influenced by trees through increased SOM input (Fig. 2, Table 2). As expected, soil temperature and moisture were negatively correlated. *R*_*s*_ was neither affected by microbial biomass, bacterial and fungal richness, SOM or tree influence (Fig. 2).

## Discussion

Overall, our results show that forest fragmentation promotes increased enzymatic activities and respiration in soil. These patterns can be explained by the direct and indirect effects of matrix influence on tree size. Therefore, our work suggests that in Mediterranean holm oak forests, the direct effect of fragmentation and agricultural matrix over tree growth triggers a complex cascade of interconnected causal-effect relations that ultimately modifies the soil environment affecting its microbial diversity and functioning as well as soil CO_2_ emissions.

Our in-depth study of possible underlying mechanisms shows how controls of microbial taxonomic diversity, microbial functions and overall biogenic soil CO_2_ emissions could be decoupled in these habitats subjected to such large human-made perturbations. For instance, microbial richness was mainly controlled by perturbations associated with the agricultural matrix influence. The lack of a relationship between the structure of the soil microbial communities (both bacterial and fungal richness) and soil enzymatic activity suggests that microbial biomass can be more relevant than microbial identity to explain these particular enzymatic activities, at least under our experimental conditions. The functional redundancy of the microbial communities, which promotes functional overlaps could be explaining this lack of relation (Curiel Yuste et al., 2014).

As previously observed in the study area, main effects of matrix influence were related to nutrient input. Large differences between soils from agricultural matrix and forest fragments promotes nutrient availability in forest edges and small fragments (Flores-Rentería et al., 2015; Flores-Rentería et al., 2016; Flores-Rentería et al., 2018). We expected a strong effect of nutrient availability on enzymatic activity since nutrients usually modulate the capacity of microbes to decompose SOM and mineralize it (Baldrian, 2014; Gómez-Luna et al., 2009; Malmivaara-Lämsä et al., 2008). However, nutrient load did not have a significant effect on enzymatic activity, which lead to a weaker influence than expected of the agricultural matrix over the enzymes activity (total effects = 0.13). Regarding specific enzymatic activities, only the *β*-glucosidase activity was, to some extent, directly and positively affected by the environmental changes associated with the agricultural matrix. This suggests that this enzyme might be affected by other quantitative processes not measured in this study, that could better reflect the classification of matrix influence e.g., changes in the quality and composition of SOM, or presence of secondary metabolites that may affect the soil system (Kuzyakov, 2010; Kuzyakov, Friedel & Stahr, 2000).

Additionally, the observed strong effect of trees over pH, which was the only environmental factor directly affecting all microbial variables (enzymatic activities, microbial richness and biomass), reinforces the idea that microbial activity is very sensitive to even small variations in soil pH (Fierer & Jackson, 2006; Sinsabaugh et al., 2008), and gives clues on mechanisms of tree/ecosystem-relation over soil biological activity. Soil pH can modify the active site conformation of enzymes, so that various enzyme isoforms can differentially perform in terms of efficiency (Frankenberger & Johanson, 1982). The net negative effect of tree size over pH (acidification) was likely explained by increasing the presence of humic acids when SOM accumulates (You, Yin & Allen, 1999).

Regarding soil respiration, our results show that fragmentation effects were modulated by tree size, which modified soil microclimatic conditions (temperature and moisture) (Curiel Yuste et al., 2003; Schlesinger & Andrews, 2000). Trees modulate climate, for example by down-regulating soil temperature through canopy radiation interception, limiting the soil water evaporation. Additionally, the SOM accumulation around trees usually increases the availability of nutrients and the capacity of soils to retain water, further buffering soil temperature, and hence decreasing water evaporation rates (Abu-Hamdeh, 2001; Hastwell & Morris, 2013). Furthermore, the higher accumulation of SOM under larger trees of small fragments (i.e., with high agricultural matrix influence) directly and positively affected soil microbial biomass also by the improvement of the environmental abiotic conditions for microbial growth (Barba et al., 2013; Pérez-Izquierdo et al., 2017). It is noteworthy that although we designed an SEM model to explain the spatial variation of *R*_*s*_, this explanatory capacity was still limited with respect to the predicted variability obtained for the enzymatic activities (18.7%, versus 82% of the variability, respectively). This is probably because *R*_*s*_, integrates not only heterotrophic (microbial) but also autotrophic (roots and mycorrhizas) metabolic activity and the latter is controlled by different variables in relation to above ground C cycle processes (Heinemeyer et al., 2007) that can greatly hinder the spatial interpretation of this flux (Barba et al., 2013; Søe & Buchmann, 2005).

## Conclusions

In line with our recent findings of a strong influence of tree canopy cover on the microbial functioning under fragmented habitats (Flores-Rentería et al., 2015; Flores-Rentería et al., 2016), this study adds a mechanistic dimension showing insights on how trees modify their environment to optimize soil functioning. Hence, anthropogenic transformation of the landscape (fragmentation) and its effect on tree growth triggers a cascade of causal-effect relations producing substantial changes in soil functioning and soil CO_2_ emissions rising from the capacity of trees to modulate the environmental conditions for both soil autotrophic and heterotrophic activities. In this respect, our study unveils key mechanisms that regulate the soil system functioning in fragmented landscapes, revealing how trees affect (either directly or indirectly) microclimate and other biotic and abiotic environmental soil variables such as pH, microbial biomass and SOM. Together, our results point out that forest fragmentation and subsequent agricultural practices may have a profound impact over the complexity of plant-soil interactions, altering the capacity of soils to sequester C and retain essential nutrients. These effects should therefore be taken into account to improve current estimations of soil C dynamics under global change scenarios.

##  Supplemental Information

10.7717/peerj.5857/supp-1 Supplemental Information 1 Supplementary material Figure S1. Contour graph of the tree influence index (*y*-axis), the distance from the trunk (*x*-axis) and the basal area measured at 25 cm from the ground (D25) (*z*-axis; cm^2^).
Figure S2. Principal Component Analysis (PCA) of soil nutrients (scores and eigenvectors) from three levels of forest fragmentation under canopy and open areas in a Holm-oak forests in Spain. Coverage is represented by different colors: gray, under canopy (UC); white, open areas (OA). Soil provenances are represented by different symbols: squares, forest interior of large fragments (Fi); circles, forest edge of large fragments (Fe); triangles, small fragments (Sf). Error bars represent standard error (taken from Flores-Rentería et al., 2016).
Table S1. Tree cover and matrix influence effects in soil metabolism, biotic and abiotic properties and tree characteristics, of Holm oak forest fragments in Spain (one-way ANOVA). Tree cover, *n* = 45 and matrix influence, *n* = 30. Significant effects (*p* < 0.05) are noted in bold. MI, Matrix influence. C, tree cover. *R*_*s*_, soil respiration. SOM, soil organic matter.

10.7717/peerj.5857/supp-2 Data S1 Raw data of habitat fragmentation

## Additional Information and Declarations

### Data Availability

The following information was supplied regarding data availability: The raw data are provided in the Supplemental File.
