# Supplementary material for: Habitat fragmentation is linked to cascading effects on soil functioning and CO2 emissions in Mediterranean holm-oak-forests
Source: PeerJ. 2018 Oct 30;6:e5857. doi: 10.7717/peerj.5857 (PMC6214227; doi:10.7717/peerj.5857)
Supplement: Supplemental Information 1 — Figure S1. Contour graph of the tree influence index (y-axis), the distance from the trunk (x-axis) and the basal area measured at 25 cm from the ground (D25) (z-axis; cm2). Figure S2. Principal Component Analysis (PCA) of soil nutrients (scores and eigenvectors) from three levels of forest fragmentation under canopy and open areas in a Holm-oak forests in Spain. Coverage is represented by different colors: gray, under canopy (UC); white, open areas (OA). Soil provenances are represented by different symbols: squares, forest interior of large fragments (Fi); circles, forest edge of large fragments (Fe); triangles, small fragments (Sf). Error bars represent standard error (taken from Flores-Rentería et al., 2016). Table S1. Tree cover and matrix influence effects in soil metabolism, biotic and abiotic properties and tree characteristics, of Holm oak forest fragments in Spain (one-way ANOVA). Tree cover, n = 45 and matrix influence, n = 30. Significant effects (p < 0.05) are noted in bold. MI, Matrix influence. C, tree cover. Rs, soil respiration. SOM, soil organic matter. [file peerj-06-5857-s001.pdf]

Habitat fragmentation is linked to cascading effects on soil functioning and CO_2 emissions in Mediterranean holm-oak-forests

Dulce Flores-Rentería[a,b*], Ana Rincón[c], Teresa Morán-López[b,d], Ana-Maria Hereş[e,f], Leticia Pérez-Izquierdo[c], Fernando Valladares[b,g], Jorge Curiel Yuste[f,h]

[a] CONACYT- CINVESTAV Unidad Saltillo, Group of Sustainability of Natural Resources and Energy. Ramos Arizpe, Coahuila, Mexico.

[b] Department of Biogeography and Global Change, Museo Nacional de Ciencias Naturales (MNCN), Spanish Scientific Council (CSIC). Madrid, Spain.

[c] Department of Soil, Plant and Environmental Quality, Instituto de Ciencias Agrarias (ICA), Spanish Scientific Council (CSIC). Madrid, Spain.

[d] Ecotono lab, INIBIOMA, CRUB, Univ. Nacional del Comahue, Bariloche, Río Negro, Argentina.

[e] Department of Forest Sciences, Transilvania University of Brasov. Brasov, Romania

[f] BC3-Basque Centre for Climate Change, Scientific Campus of the University of the Basque Country. Leioa, Spain.

[g] Department of Biology and Geology, Universidad Rey Juan Carlos. Móstoles (Madrid), Spain.

[h] IKERBASQUE. Basque Foundation for Science. Bilbao Bizkaia - Spain.

*Corresponding Author: Dulce Flores-Rentería, CONACYT- CINVESTAV Unidad Saltillo, Grupo de Sustentabilidad de los Recursos Naturales y Energía - Av. Industria Metalúrgica 1062, Parque Industrial Ramos Arizpe, Ramos Arizpe, C.P. 25900, Coahuila, México Tel: +52 844 4389625 ext 8426; e-mail: yaahid@gmail.com

Supplementary

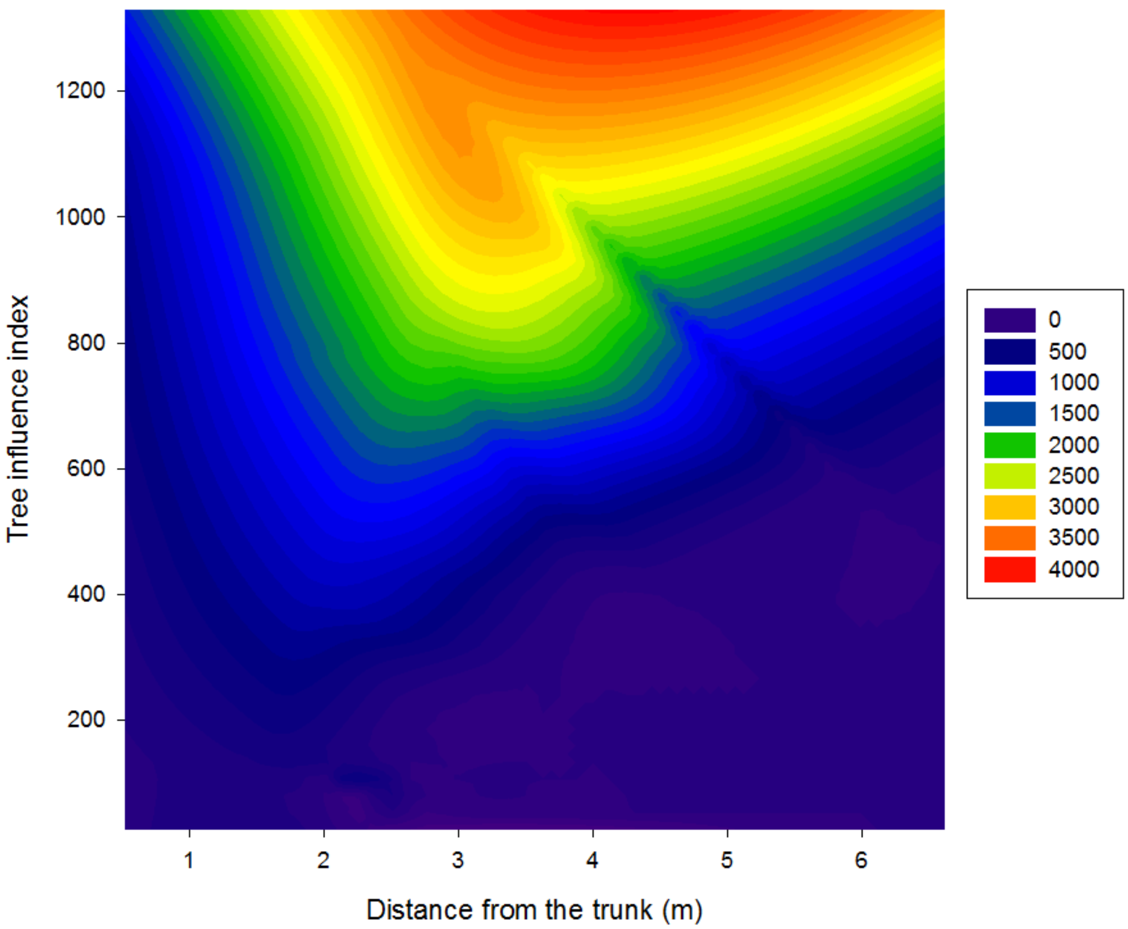

Fig. S1 Contour graph of the tree influence index (y-axis), the distance from the trunk (x-axis) and the basal area measured at 25 cm from the ground (D_{25}) (z-axis; cm^2).

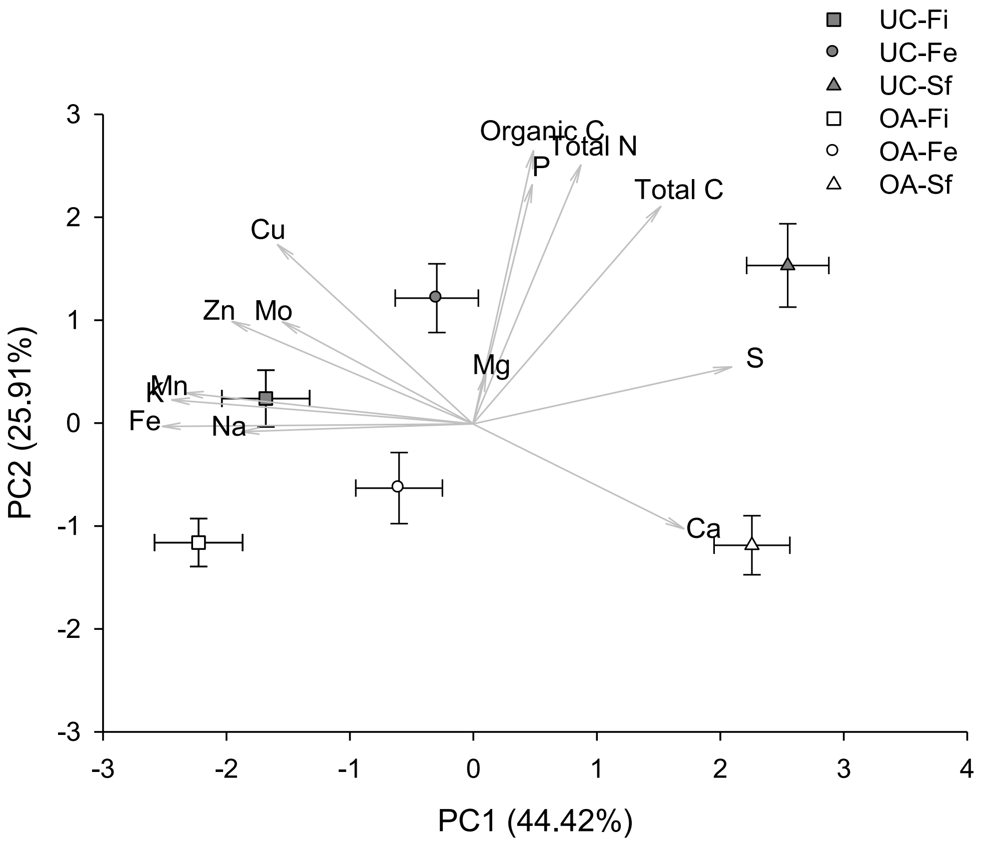

Fig. S2 Principal Component Analysis (PCA) of soil nutrients (scores and eigenvectors) from three levels of forest fragmentation under canopy and open areas in a Holm-oak forests in Spain. Coverage is represented by different colors: gray = under canopy (UC); white = open areas (OA). Soil provenances are represented by different symbols: squares = forest interior of large fragments (Fi); circles = forest edge of large fragments (Fe); triangles = small fragments (Sf). Error bars represent standard error (taken from Flores-Rentería et al., 2016).

Table S1 Tree cover and matrix influence effects in soil metabolism, biotic and abiotic properties and tree characteristics, of Holm oak forest fragments in Spain (one-way ANOVA). Tree cover, n=45 and matrix influence, n=30. Significant effects (p < 0.05) are noted in bold. MI = Matrix influence. C = tree cover. R_s = soil respiration. SOM = soil organic matter.

		**Under canopy**	**Open areas**	**Forest interior**	**Forest edge**	**Small fragments**
		**MI**	**MI**	**C**	**C**	**C**
β-glucosidase	F	**6.53**	**13.80**	**57.34**	**14.40**	**17.11**
(pmol min^{-1} mg^{-1})	*p*	**<0.001**	**<0.001**	**<0.001**	**<0.001**	**<0.001**
Chitinase	F	1.99	**4.53**	**8.37**	**12.25**	**39.00**
(pmol min^{-1} mg^{-1})	*p*	0.15	**0.02**	**0.01**	**<0.001**	**<0.001**
Phosphatase	F	**5.79**	0.42	**56.85**	**36.70**	**64.97**
(pmol min^{-1} mg^{-1})	*p*	**0.01**	0.66	**<0.001**	**<0.001**	**<0.001**
R_s	F	0.84	2.89	1.70	**4.81**	1.34
(μmol (CO_2) m^2 s^{-1})	*p*	0.44	0.07	0.20	**0.04**	0.26
SOM	F	1.81	0.89	**28.99**	**21.93**	**14.62**
(%)	*p*	0.18	0.42	**<0.001**	**<0.001**	**<0.001**
pH	F	0.75	1.19	**12.89**	**7.50**	**7.78**
	p	0.48	0.31	**<0.001**	**0.01**	**0.01**
Soil moisture	F	1.27	**4.93**	**35.62**	**4.57**	**19.52**
(%)	*p*	0.29	**0.01**	**<0.001**	**0.04**	**<0.001**
Soil temperature	F	**3.41**	**7.13**	**129.08**	**23.97**	**120.26**
(ºC)	*p*	**0.04**	**<0.001**	**<0.001**	**<0.001**	**<0.001**
Tree basal area	F	**8.79**	-	-	-	-
(cm^2)	*p*	**<0.001**	-	-	-	-
Tree height	F	**10.09**	-	-	-	-
(m)	*p*	**<0.001**	-	-	-	-
Tree canopy	F	**12.02**	-	-	-	-
(m^2)	*p*	**<0.001**	-	-	-	-
Tree influence index	F	**5.08**	**5.80**	**29.01**	**23.77**	**12.58**
	p	**0.01**	**0.01**	**<0.001**	**<0.001**	**<0.001**
Bacterial richness	F	**4.36**	**3.93**	0.66	2.34	0.30
(S)	*p*	**0.019**	**0.027**	0.123	1.38	0.59
Fungal richness	F	1.34	0.73	0.081	0.50	1.57
(S)	*p*	0.26	0.49	0.77	0.49	0.22
Microbial biomass	F	2.42	1.47	**31.65**	**28.15**	**13.83**
(mg C kg^{-1})	*p*	0.10	0.24	**<0.001**	**<0.001**	**<0.001**
